# Nanoceria Particles Are an Eligible Candidate to Prevent Age-Related Macular Degeneration by Inhibiting Retinal Pigment Epithelium Cell Death and Autophagy Alterations

**DOI:** 10.3390/cells9071617

**Published:** 2020-07-04

**Authors:** Annamaria Tisi, Vincenzo Flati, Simona Delle Monache, Luca Lozzi, Maurizio Passacantando, Rita Maccarone

**Affiliations:** 1 Department of Biotechnology and Applied Clinical Sciences, University of L’Aquila, via Vetoio, Coppito 2, 67100 L’Aquila, Italy; annamaria.tisi@graduate.univaq.it (A.T.); vincenzo.flati@univaq.it (V.F.); simona.dellemonache@univaq.it (S.D.M.); 2 Department of Physical and Chemical Sciences, University of L’Aquila, via Vetoio, Coppito 1, 67100 L’Aquila, Italy; luca.lozzi@univaq.it (L.L.); maurizio.passacantando@univaq.it (M.P.)

**Keywords:** nanomedicine, nanoceria, retinal pigment epithelium, light damage, autophagy, atrophic AMD

## Abstract

Retinal pigment epithelium (RPE) dysfunction and degeneration underlie the development of age-related macular degeneration (AMD), which is the leading cause of blindness worldwide. In this study, we investigated whether cerium oxide nanoparticles (CeO_2_-NPs or nanoceria), which are anti-oxidant agents with auto-regenerative properties, are able to preserve the RPE. On ARPE-19 cells, we found that CeO_2_-NPs promoted cell viability against H_2_O_2_–induced cellular damage. For the in vivo studies, we used a rat model of acute light damage (LD), which mimics many features of AMD. CeO_2_-NPs intravitreally injected three days before LD prevented RPE cell death and degeneration and nanoceria labelled with fluorescein were found localized in the cytoplasm of RPE cells. CeO_2_-NPs inhibited epithelial-mesenchymal transition of RPE cells and modulated autophagy by the down-regulation of LC3B-II and p62. Moreover, the treatment inhibited nuclear localization of LC3B. Taken together, our study demonstrates that CeO_2_-NPs represent an eligible candidate to counteract RPE degeneration and, therefore, a powerful therapy for AMD.

## 1. Introduction

Retinal pigment epithelium (RPE) is a monolayer of post-mitotic, non-regenerating epithelial cells which, together with the choriocapillaris and Bruch’s membrane, constitutes the outer blood retinal barrier (BRB) structure [1,2]. At their apical surface, RPE cells are connected through tight junctions, which allow them to form a barrier. This leads to a size-selective passive diffusion of molecules [3]. Hence, RPE integrity is fundamental for the physiological maintenance of the BRB. Moreover, RPE cells are essential to provide nutrients to the neuroretina for vitamin A metabolism, for shedding the photoreceptor outer segments (POS), and, therefore, for the visual cycle [4]. Given the fundamental role of the RPE in retinal homeostasis, its dysfunction and cell death are key factors involved in the development of several retinal disorders, including age-related macular degeneration (AMD) [5], which is the leading cause of blindness in advanced age [6,7]. In particular, the dry form of AMD is characterized by progressive lipofuscin/drusen accumulation associated with the slow apoptosis of RPE, neuroretina, and choriocapillaris and, in the end, with permanent central vision loss [8,9]. In early dry AMD, RPE cells assume a mesenchymal morphology, which is a process known as the epithelial-mesenchymal transition (EMT) [10,11,12,13,14] while photoreceptors appear intact. With progressive RPE changes and degeneration, patients develop the advanced form of age-related macular degeneration, called geographic atrophy (GA), characterized by the development of an extended atrophic retinal region in the macula [15]. As a consequence, RPE cells die and the downstream photoreceptors are compromised, cannot work correctly, and degenerate [16,17]. Thus, the visual process is altered. The molecular mechanisms underlying the RPE dysfunction and death are not yet fully understood. Among these, autophagy is considered a key mechanism for RPE cells’ homeostasis maintenance [18] and may be implicated in AMD development [5,19,20,21]. The physiological functions of autophagy rely on the removal of damaged organelles and proteins [22]. This is particularly important in RPE cells due to their high metabolic demand and due to the active POS phagocytosis [23]. However, controversial data indicate that both activation and inhibition of autophagy in RPE cells could play either a protective or damaging role under stress conditions in AMD patients [21,24,25]. It has been shown that impaired autophagy is involved in a defective clearance of metabolites in RPE, which leads to lipofuscin accumulation [26]. Other studies have shown that the up-regulation of autophagy is involved in the crosstalk with cell death pathways [21,27]. Recently, it has also been demonstrated that autophagy dysregulation could be a mechanism underlying the EMT process [28]. It has been proposed that autophagy induced-EMT could be mediated by an increased oxidative stress [29], which is a common condition found in patients suffering from AMD [14,26]. Accordingly, the oxidative stress is considered a major activator of autophagy in several tissues, including the retina [30]. On this basis, targeting oxidative stress could prevent autophagy activation and RPE dysfunction with subsequent retinal neuroprotection. We have previously demonstrated that light damage induced oxidative stress, which was reduced in the photoreceptor layer by the antioxidant activity of cerium oxide nanoparticles. This leads to the neuroretina protection [31,32,33,34]. Cerium Oxide nanoparticles (CeO_2_-NPs or nanoceria) are anti-oxidant agents, which show peculiar physical-chemical features, as they have auto-regenerative properties without exhaustion [35], and are retained in the retina for a long time after intravitreal injection [34]. In our previous studies, nanoceria labelled with fluorescein-isothiocyanate (FITC-CeO_2_) have been found in the outer retina in a region that includes photoreceptor outer segments and RPE cells [33,34], suggesting that the outer retina could be the target of nanoceria particles by triggering RPE protection as the main mechanism. However, until now, the effects of cerium oxide nanoparticles on the RPE have never been investigated. This is an important issue to be addressed since the preservation of the RPE is the first step necessary to slow down the degeneration processes and protect visual function in AMD patients. Moreover, to date, no treatments exist to effectively protect RPE cells in patients with dry AMD. Hence, they are often inevitably destined to blindness [36]. Therefore, in order to improve the management of AMD, we investigated whether the RPE was structurally preserved by nanoceria particles’ intravitreal injection in the acute light damage (LD) experimental model. The acute LD is a well-known animal model of both dry [37] and wet AMD [38] and allows one to effectively study protective strategies, which target the RPE. The light exposure leads to oxidative stress, autophagy alterations, EMT induction in the RPE, and retinal degeneration [39] as well as in AMD patients. The effects of nanoceria particles on photo-oxidation-induced autophagy in the RPE were also investigated. Identifying this way of action of the nanoceria particles is fundamental in order to attribute them a therapeutic value for the atrophic form of AMD.

## 2. Materials and Methods

### 2.1. Cell Culture

Adult retinal pigment epithelial cells (ARPE-19) were purchased from the American Type Culture Collection (ATCC, Manassas, VA, USA), seeded (5 × 10^5^ cell/density) into a 25-cm^2^ flask, and cultured in a 1:1 mixture of Dulbecco’s Modified Eagle Medium (DMEM) and Ham’s F12 medium with 10% Fetal Bovine Serum (FBS, EuroClone, Pero (MI), Italy), 1% Glutamine, and 1% penicillin and streptomycin. The cells were maintained in a 37 °C incubator in a humidified atmosphere containing 5% CO_2_, and passaged twice weekly. In places not otherwise specified, the reagents were acquired from Euroclone, Pero (MI), Italy.

### 2.2. H_2_O_2_ and Nanoceria Treatment

ARPE-19 cells (fourth passage) were seeded at 5 × 10^4^ cells/mL into 24-well plates and grown for 48 h to sub-confluence. The cells were treated with 200 to 1000 µM hydrogen peroxide (H_2_O_2_, Sigma, St. Louis, MO, USA) in the culture media for up to 24 h as required. For the control group, the cells were left untreated in culture medium.

Nanoceria were synthesized using a mixture of Ce(NO_3_)_3_·6H_2_O and ethylene glycol was stirred for 30 min, and then added with ammonium hydroxide (NH_4_OH). The obtained product was calcined in an air furnace at 500 °C. More details about the procedure and structural and electronic properties were previously described [35].

In order to evaluate cell proliferation, the following conditions were used: untreated control, hydrogen peroxide at increasing concentrations (200 μM, 400 μM, 600 μM, 800 μM and 1000 μM) with or without nanoceria, and nanoceria alone. Cerium oxide nanoparticles were suspended in DMEM:F12 at the 1 mM concentration. The cells were incubated at 37 °C, 5% CO_2_ in a humidified atmosphere for 24 h.

### 2.3. Cell Proliferation Assay

Cell proliferation was evaluated by fixing cells with formalin 4% and staining with crystal violet (1%). Stained cells were solubilized using a solubilization solution containing 1% SDS and 50% methanol and the reading was carried out on 96-well multi-well (EuroClone, Milan, Italy) plates in a microplate reader at 595 nm.

### 2.4. Animal Model

All experiments were conducted in accordance with the ARVO statement for the use of animals in ophthalmic and vision research, authorization number 448/2016-PR by the Italian Ministry of Health. Sprague-Dawley albino rats were used as an animal model. Animals were born and raised in dim cyclic light conditions (12 h light, 12 h dark) with an ambient light level of approximately 5 lux.

### 2.5. Light Damage

In order to induce retinal degeneration and study the progression of RPE changes with light exposure, albino rats were dark-adapted and then exposed to bright light (1000 lux) for different times: 6 h, 12 h, and 24 h. One group was recovered for 7 days at 5 lux in dim cyclic conditions after 24 h of light exposure. To perform light damage (LD), the animals were placed in individual plexiglass cages with food and water, as previously described [33,34]. Light exposure always started at 9 a.m. in order to not alter the circadian rhythm.

### 2.6. Nanoceria Intravitreal Injection

The rats were anaesthetized with an intraperitoneal injection of Ketamine/Xylazine (10 mg/100 g–1.2 mg/100 g) and 2 μL of cerium oxide nanoparticles (1 mM, suspended in NaCl 0.9%) were intravitreally injected in both eyes of albino rats using a Hamilton syringe under total sterile conditions. At the end of the injection procedure, a drop of ophthalmic antibiotic (Tobral 0.3%) was applied on the eyes to prevent post-surgical infections. The animals were then returned to their cages, monitored to ensure their complete awakening and good health, and housed in the animal room in dim cyclic conditions for three days before undergoing light damage. Based on the analysis of the LD time course, we decided to observe the effects of the nanoceria treatment immediately after LD, which is when the RPE layer was not destroyed but apoptosis and autophagy disturbances were already detectable in the RPE cells. We also investigated the effects of the treatment 7 days after LD, which is when the RPE layer was destroyed and, therefore, allowed us to better understand the degree of RPE protection exerted by the nanoceria.

The experimental groups of the study are listed below and their sample size is indicated in the figure captions.

CTRL: healthy rats

Light damage:

LD6h: light exposure (1000 lux) for 6 h

LD12h: light exposure (1000 lux) for 12 h

LD24h: light exposure (1000 lux) for 24 h

LD24h + 7 rec: light exposure (1000 lux) for 24 h followed by 7 days of recovery (5 lux)

Nanoceria treatment:

CeO_2_ + LD24h: intravitreal injection of nanoceria [1 mM] + LD24h

CeO_2_ + LD24h + 7 rec: intravitreal injection of nanoceria [1 mM] + LD24h followed by 7 days of recovery

### 2.7. Retinal Cryosections

At the end of each LD time, the animals were euthanized and the eyes enucleated for morphological analyses. The eyes were enucleated and fixed in 4% paraformaldehyde for 6 h and washed in 0.1 M phosphate buffered saline (PBS, pH 7.4). After the eye-cup processing, the eyes were cryoprotected by immersion in different sucrose concentrations (10%, 20%, and 30%) embedded in the compound OCT (optimum cutting temperature) (Tissue Tek, Qiagen, Hilden, Germany) and frozen. Cross sections of 20-μm thickness were made for each retina and collected on gelatine and poly-l-lysine-coated slides for subsequent analyses. In order to compare the different experimental groups correctly, the retinal sections crossing the optic nerve were analyzed.

### 2.8. Cryosections Analysis

#### 2.8.1. Immunostaining

Cryosections were used for immunostaining in order to visualize the RPE and detect the autophagosomes localization. 5% BSA (bovine serum albumin) was used to block non-specific bindings. Sections were then incubated overnight at 4 °C with primary antibodies: anti-RPE65 (Abcam, Cambridge, UK) (1:250 in 1% BSA) and anti-LC3B (Cell Signaling, Danver, CO, USA) (1:250 in 1% BSA) for retinal pigment epithelium and autophagosomes detection, respectively. Secondary antibodies were: anti-rabbit IgG conjugated to green fluorescent dye (Alexa Fluor 488, Molecular Probes, Invitrogen, Carlsbad, CA, USA) for anti-LC3B and anti-mouse IgG conjugated to red fluorescent dye (Alexa Fluor 594, Molecular Probes, Invitrogen, Carlsbad, CA, USA) for anti-RPE65. Secondary antibodies were diluted 1:1000 in Phosphate Buffered Saline (PBS 1X) and incubated at 37 °C for 2 h. Confocal images were acquired, by setting up the same parameters, using a Leica TCS SP5 confocal microscope.

#### 2.8.2. TUNEL Assay

The cryosections were also used for the TUNEL (deoxynucleotidyl transferase d-UTP nick end labelling) assay, together with anti-RPE65 and anti-LC3B immunostaining, in order to quantify dying cells in the RPE and to identify the localization of the autophagosomes in the dying RPE cells. The TUNEL assay was performed as described previously [40]. At the end of the procedure, TUNEL positive nuclei were detected in red. Anti-RPE65 and anti-LC3B immunostainings were performed as described above. Instead, the secondary antibody used for anti-RPE65 was an anti-mouse IgG conjugated to green fluorescent dye (Alexa Fluor 488, Molecular Probes, Invitrogen, Carlsbad, CA, USA). Dying RPE cells were counted along the entire cryosections from a superior to an inferior edge crossing the optic nerve. The Nikon Eclipse 80i fluorescence microscope or the Leica TCS SP5 confocal microscope were used to acquire the images.

### 2.9. Western Blot

Eyecups were also used for protein analysis through a Western blot. Immediately after sacrifice, lens and cornea were removed from the eye and the eye cups were frozen and stored at −80 °C. For protein extraction, eye cups were homogenized in a lysis buffer (50 mM Tris.Cl pH 7.8, 1% Triton X100, 0.1% SDS, 250 mM NaCl, 5 mM EDTA, 100 mM NaF, 2 mM NaPPi, 2 mM Na3VO4, 1 mM PMSF, 1mM Aprotinin, 1mM Pepstatin, 1mM Leupeptin) on ice by using a Dounce homogenizer. The samples were centrifuged and the soluble phase was recovered and used for the subsequent analysis. Protein concentration was quantified by using the Bradford assay (Bio-Rad Laboratories, Milan, Italy), and 70 μg of total protein extracts were run on a Bolt 4–12% Bis-Tris Plus (Thermo Fisher Scientific, Waltham, MA, USA) at 200 V for 20 min. The proteins were transferred to a Polyvinylidene fluoride (PVDF) membrane (Millipore, Milan, Italy) through the iBlot 2 Dry Blotting System (Invitrogen IB21001). Membranes were blocked with 5% of blotting grade milk in Tris-Buffered Saline containing 0.1% Tween20 (TBST). Specific proteins were detected with the following primary antibodies diluted in 5% non-fat dry milk in TBST: rabbit anti-LC3B (Cell Signaling, Danvers, CO, USA) (1:1500), anti-p62 (Cell Signaling, Danvers, CO, USA) (1:1000), anti-tubulin (Santa Cruz Biotechnology, Heidelberg, Germany), and tubulin (Thermo Scientific, Waltham, MA, USA)). Secondary antibodies were anti-rabbit or anti-mouse (depending on the primary antibody) Horseradish Peroxidase (HRP)-conjugated mixture (Bio-Rad Laboratories, Milan, Italy) diluted 1:2000 in TBST containing 5% non-fat milk. The membranes were developed with SuperSignal West Pico chemiluminescent substrate (Thermo Fisher Scientific Inc., Rockford, IL, USA). The protein bands were detected using a BioRad ChemiDoc XRS-plus imaging system (Bio-Rad Laboratories, Milan, Italy). Densitometric analysis was conducted by using the ImageJ software (U.S. National Institutes of Health, Bethesda, MD, USA) and the amount of proteins was normalized versus tubulin.

### 2.10. Flat Mounted RPE

Immediately after animal sacrifice, the eye cup procedure was performed. The retina was gently removed from the eye cup. The RPE-choroidal complex was left intact in eye cup samples for flat mounted (FM) RPE. Eye cups were then fixed in 4% paraformaldehyde for 1 h, washed three times in 0.1 M phosphate buffered saline (PBS, pH 7.4), and then processed for subsequent analysis. Eye cup samples were used to analyse the RPE structure. Briefly, non-specific bindings were blocked with 5% BSA for 1 h. Eye cups were then labelled with Phalloidin–FITC (Fluorescein Isothiocyanate) conjugated (Sigma Aldrich, Saint Louis, MO, USA) (1:250 in PBS) overnight at 4 °C to detect cytoskeleton organization and cell edges. Afterward, the eye cups were immunolabeled with primary antibody anti-RPE65 (Abcam, Cambridge, UK) (1:250 in 1% BSA) overnight at 4 °C. The secondary antibody was an anti-mouse IgG conjugated to red fluorescent dye (Alexa Fluor 594, Molecular Probes, Invitrogen, Carlsbad, CA, USA), diluted 1:200, and incubated at 37 °C for 2 h. Lastly, the eye cups were counterstained with nuclear staining Bisbenzimide (Hoechst) and collected on gelatine and poly-l-lysine-coated slides for subsequent observations.

All the images were acquired through a Leica TCS SP5 confocal microscope.

### 2.11. Statistical Analysis

Student’s *t*-test and the one-way ANOVA test were used to perform the statistical analysis. First type error was set at 5%. Post-hoc comparisons were adjusted using Tukey’s test. The statistical analysis was conducted by using the SigmaPlot 12.0 software.

## 3. Results

### 3.1. Nanoceria Attenuate H_2_O_2_-Induced Cytotoxicity in ARPE-19 Cells

In order to evaluate the protective effects of cerium oxide nanoparticles against H_2_O_2_-induced cytotoxicity, ARPE-19 cells were treated with increasing concentrations of H_2_O_2_ (200 µM, 400 µM, 600 µM, 800 µM, and 1000 µM) for 24 h and the degree of cell proliferation was evaluated by Crystal violet staining. Our results showed that hydrogen peroxide produced a progressive cytotoxic effect in the ARPE-19 cells with the increase of H_2_O_2_ concentration, as highlighted in other studies [41,42]. The cytotoxic effect was significant at 600 µM with 35% toxicity and reaching a maximum effect at 1000 µM with a toxicity of about 60% (Supplementary Materials, Figure S1). The untreated cells (CTRL) did not show any sign of cell death even after 24 h, which indicates that H_2_O_2_ induced cell death in a dose-dependent manner in this culture system.

**Figure 1:**
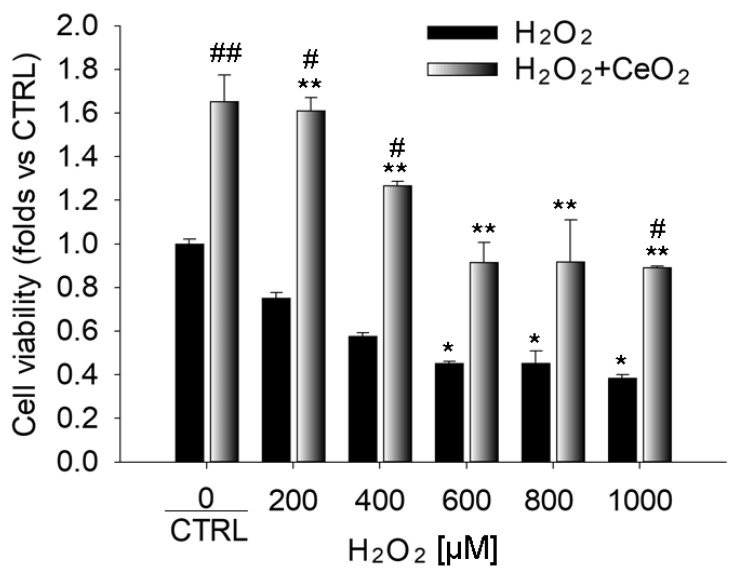
Protection of ARPE-19 cells from H_2_O_2_-induced toxicity by nanoceria. Cell viability assay obtained with crystal violet staining on ARPE-19 cells stressed with H_2_O_2_ at increasing concentrations (200 µM, 400 µM, 600 µM, 800 µM, and 1000 µM) with or without cerium oxide nanoparticles (1 mM). Data are shown as mean ± SE. Statistical analysis was performed by one-way ANOVA and followed by the Tukey test (*n* = 4). * *p* < 0.05, ** *p* < 0.005, *** *p* < 0.0001 versus CTRL, ^#^ *p* < 0.05, ^##^ *p* < 0.005, ^###^ *p* < 0.0001 versus H_2_O_2_.

Next, we aimed to evaluate whether nanoceria treatment could protect ARPE-19 cells from H_2_O_2_- induced cell death. The nanoparticles (CeO_2_-NPs; 1 mM) reversed the H_2_O_2_-induced reduction of cell viability in a dose-dependent manner. In the presence of CeO_2_-NPs, the cell viability of ARPE-19 cells treated with hydrogen peroxide was increased in all experimental conditions (Figure 1), which reverted the cytotoxic effect of H_2_O_2_ and aligned the growth levels of ARPE-19 co-treated with CeO_2_-NPs to that of the CTRL. Cerium oxide nanoparticles added to the culture medium allowed an increased proliferation rate of ARPE-19 cells when compared to the control, which suggests that they can act to promote cell survival in the absence of cytotoxic stimuli.

### 3.2. Nanoceria Localize in the RPE after Intravitreal Injection

Previous evidence showed that cerium oxide nanoparticles can cross the inner limiting membrane and reach the outer retina after intravitreal injection. Cerium oxide nanoparticles were found localized in the region, which includes the photoreceptors’ outer segments (OS) and the RPE [43]. Moreover, after a single administration, they remained at the same localization up to two months [34]. On this basis, we supposed that cerium oxide nanoparticles could target the RPE and prevent its degeneration and, thus, exert retinal protection.

**Figure 2:**
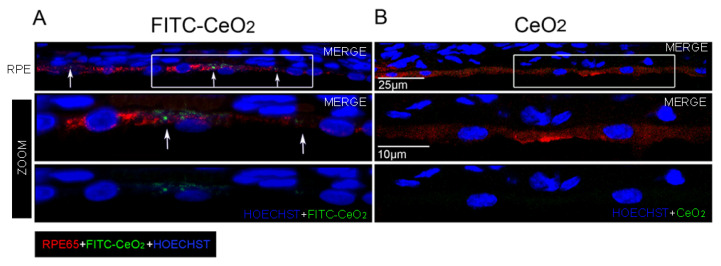
Localization of cerium oxide nanoparticles in the retinal pigment epithelium. Representative confocal images of retinal cryosections of albino rats immunolabeled with anti-RPE65 (red) in order to detect the retinal pigment epithelium: (**A**) intravitreally injected with fluorescein-isothiocyanate (FITC-CeO_2_) (green), the white arrows indicate the FITC-CeO2 agglomerates, which localize in the retinal pigment epithelium (RPE); (**B**) Intravitreally injected with standard cerium oxide nanoparticles. The high magnifications show the regions highlighted in the white frames. CeO_2_: cerium oxide nanoparticles. FITC-CeO_2_: cerium oxide nanoparticles labeled with FITC.

To confirm our hypothesis, we intravitreally injected nanoceria labeled with FITC (FITC-CeO_2_), obtained as reported in Fiorani et al., 2015 [33], into the rats’ eyes and marked the RPE by immunostaining for RPE65 protein, which is a selective marker of retinal pigment epithelium (Figure 2). Through confocal microscopy, we found that cerium oxide nanoparticles were localized in the cytoplasm of RPE cells in the form of agglomerates with different sizes (Figure 2A). In fact, due to their nano size, cerium oxide nanoparticles can be visualized only when aggregated [43]. The presence of nanoceria in the RPE was further corroborated by observing the retinal sections of eyes intravitreally injected with the nanoceria without fluorescent labeling (Figure 2B). By using the same confocal microscope setup for image acquisition used to detect FITC-CeO2, a green auto-fluorescent signal was not revealed.

### 3.3. Nanoceria Prevent RPE Degeneration

**Figure 3:**
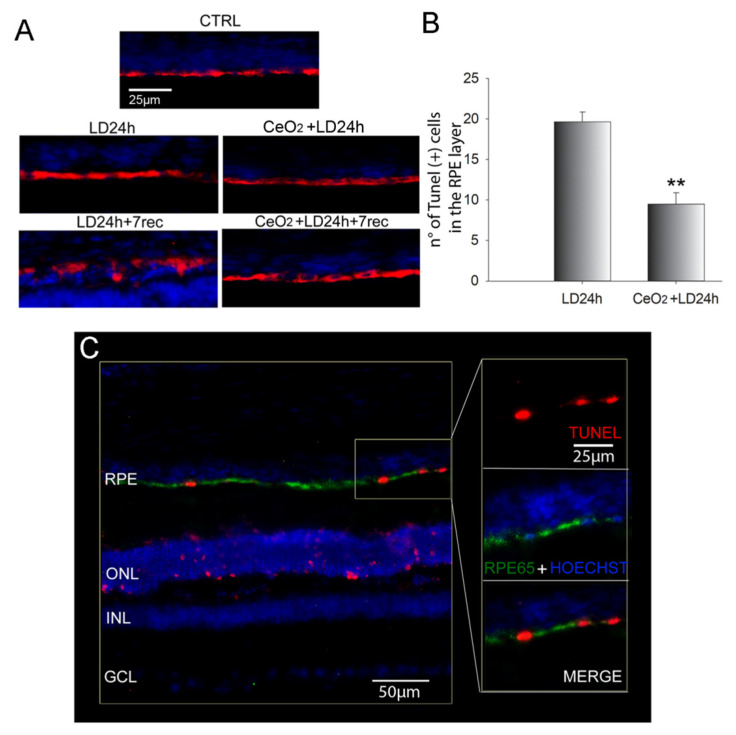
Analysis of Retinal Pigment Epithelium (RPE) degeneration after LD. (**A**) Representative images of anti-RPE65 (red) immunolabeled retinal cryosections counterstained with Hoechst (blue) and acquired by a fluorescence microscope. Scale bar: 25 µm. (**B**) Comparison of the number of TUNEL (+) cells in the RPE layer between treated and untreated animals at 24 h of light exposure. Data are shown as mean ± SE. Statistical analysis was performed by Student’s *t*-test. (*n* = 6) ** *p* < 0.01 versus LD24h. (**C**) Representative fluorescence image of a retinal cryosection after 24 h of LD, showing how TUNEL (+) cells were identified to count them. On the right side, a higher magnification of the RPE. In red TUNEL (+) nuclei, in green anti-RPE65, in blue nuclei stained with bisbenzimide. Scale bars: 50 µm (left) and 25 µm (zoom). CTRL: control. LD6h: animals exposed to 6 h of light damage. LD12h: animals exposed to 12 h of light damage. LD24h: animals exposed to 24 h of light damage. CeO_2_ + LD24h: animals treated with cerium oxide nanoparticles and exposed to 24 h of light damage. CeO_2_ + LD24h + 7 rec: animals treated with cerium oxide nanoparticles and exposed to 24 h of light damage followed by 7 days of recovery. RPE: retinal pigment epithelium. ONL: outer nuclear layer. INL: inner nuclear layer. GCL: ganglion cell layer.

It is known that photo-oxidative damage causes RPE degeneration [39]. Hence, the specific nanoceria localization in the RPE cells (Figure 2) suggests that the RPE could be the main target of cerium oxide nanoparticles, which mediates the subsequent photoreceptor neuroprotection from light damage [32]. To confirm our hypothesis, we tested whether cerium oxide nanoparticles protected the RPE in our LD experimental model. To gain this purpose, we first performed a time course analysis of RPE degeneration by analyzing anti-RPE65 immunolabeled retinal cryosections after 6 h, 12 h, and 24 h of LD and after 7 days from 24 h of LD (Figure S2A). This allowed us to determine the appropriate time points to investigate the effects of nanoceria on the RPE. The time course analysis revealed that the RPE was intact up to 24 h of light exposure while, after 7 days from LD, the RPE65 signal was altered and appeared agglomerated, which indicates that the RPE was losing its morphological structure. Therefore, we decided to evaluate RPE protection by nanoceria immediately and 7 days after LD. The RPE tissue was found to be intact in the presence of nanoceria in both cases (Figure 3A).

Dying cells in the RPE were then quantified by the TUNEL Assay together with anti-RPE65 immunostaining on retinal cryosections. Apoptotic cells were significantly increased after 12 h of LD compared to the control, with a peak at 24 h of light exposure in the central dorsal retina (Figure S2B). After 7 days from LD, the RPE was mostly degenerated because the cells were likely already dead. Therefore, it was not possible to quantify RPE65/Tunel positive cells in the dorsal retina. However, after seven days of recovery, Tunel positive RPE cells were still present at the limits of the degenerated area (not shown). The number of RPE65/Tunel positive cells was significantly reduced in the nanoceria-treated animals compared to the untreated ones immediately after LD (Figure 3B). Figure 3C shows a representative fluorescence image of how the Tunel positive RPE cells were counted.

### 3.4. Nanoceria Attenuate Light-Induced EMT in RPE Cells

**Figure 4:**
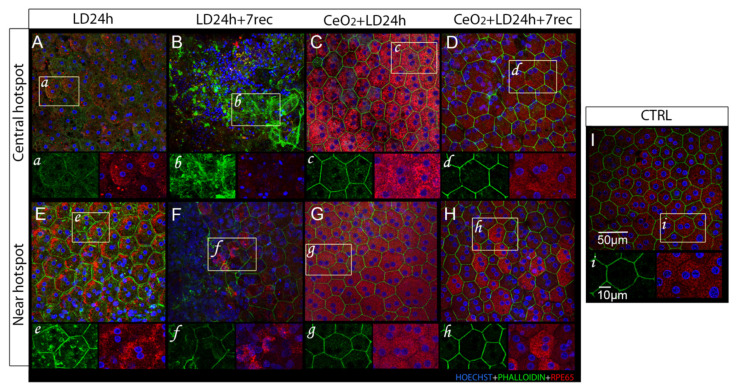
Analysis of Choroidal-RPE flat mounts. Representative confocal images of choroidal-RPE flat mounts of nanoceria-treated and untreated animals labeled with anti-RPE65 (red), phalloidin staining (green), and counter-stained with hoechst (blue). The images were acquired at the central and peripheral hotspot of the central superior retina in each experimental group. LD24h: animals exposed to light damage (1000 lux) for 24 h (**A**,**E**). LD24h + 7 rec: animals exposed to LD24h followed by seven days of recovery (**B**,**F**). CeO_2_ + LD24h: animals treated with cerium oxide nanoparticles and then exposed to LD24h (**C**,**G**). CeO_2_ + LD24h + 7 rec: animals treated with cerium oxide nanoparticles and then exposed to LD24h and let recover for seven days (**D**,**H**). Control CTRL (**I**). (**a**–**i**) show high magnifications of the RPE cells highlighted by the white frames.

Light-induced photo-toxicity triggers epithelial-mesenchymal transition (EMT) in RPE cells [39], which is a process of epithelial cells de-differentiation. This includes low or absent expression of RPE65, multinucleation, loss of the physiological cell shape and of RPE tight junctions, as well as reorganization of the cytoskeleton [44,45]. Hence, EMT implies the loss of RPE integrity and function, which culminates in its degeneration. In order to investigate whether nanoceria can prevent the EMT in the RPE and cell death, we analyzed choroidal-RPE flat mounts of treated and untreated animals at different time points after light damage (LD) (Figure 4). We focused on the “central hotspot” region, which is the area affected by light damage and present at the central superior retina. We also looked at the “peripheral hotspot” at the edge of the damaged region.

#### 3.4.1. Central Hotspot

Immediately after light exposure (Figure 4A,a), several multinucleated cells were observed at the central hotspot. The phalloidin staining appeared dispersed in the cell cytoplasm and RPE65 expression was visibly decreased compared to the control (Figure 4I,i), which suggested that those cells were undergoing a process of de-differentiation. After 7 days from LD, the RPE was completely destroyed and RPE cells were not recognizable (Figure 4B,b). Otherwise, in the nanoceria-treated animals, the RPE was markedly preserved. Both immediately (Figure 4C,c) and 7 days after LD (Figure 4D,d), the RPE cells showed the physiological hexagonal shape, although phalloidin staining was also found slightly dispersed in the cytoplasm. The RPE65 signal was clearly detectable, but it resulted in more intensity immediately after LD when compared to the control group. We also noticed that several multinucleated cells were present at the dorsal area of the nanoceria-treated animals 7 days after LD. Moreover, the nuclei were mostly placed at the periphery of the cells, which is a common sign of RPE cells’ stress [46,47].

#### 3.4.2. Peripheral Hotspot

After LD, the peripheral hotspot featured a less damaged RPE. After 24 h of LD, the cell shape was preserved and phalloidin staining delineated the cellular edges (Figure 4E,e). However, some phalloidin spots were present in the cell cytoplasm and RPE65 was found to be highly expressed and accumulated at the cell periphery. An altered morphology of the RPE cells was also observed at the edge of the degenerated area after 7 days from LD (Figure 4F,f). In fact, we detected several multinucleated cells, propagation of the phalloidin signal, heterogeneous expression of RPE65, and altered cellular shapes. These observations suggest that RPE degeneration was still propagating at the edge of the degenerated region seven days after the injury. In the nanoceria-treated animals, the RPE appeared to be even more protected than in the central hotspot region at 24 h (Figure 4G,g) and seven days after LD (Figure 4H,h). The correct hexagonal morphology of the RPE cells was maintained, as observed by phalloidin staining. RPE65 was homogeneously expressed immediately after LD (Figure 4G,g), while few cells showed a more intense RPE65 signal after seven days from LD (Figure 4H,h). Taken together, these observations indicate that nanoceria treatment prevented the occurrence of light-induced EMT in the RPE, which presented only few signs of stress, and effectively protected the RPE from degeneration up to seven days from LD.

### 3.5. Autophagy Alterations Are Prevented by Nanoceria

It has been demonstrated that light damage induces the up-regulation of autophagy and that its suppression protects the retina from degeneration [25]. Moreover, recent studies suggested that autophagy alterations could be involved in EMT of RPE cells [28]. On this basis, we wondered whether the protection of the RPE and the inhibition of EMT features by cerium oxide nanoparticles were associated with the modulation of autophagy. To answer this question, we analysed the amount of LC3B-II and p62 proteins. The first one is a common marker of autophagy activation since it is produced in the cells when the autophagosomes are generated. The levels of LC3B-II do not indicate that the autophagy process has reached completion. To complete the degradation of the engulfed material, the fusion between autophagosomes and lysosomes should occur. This process can be monitored through the analysis of the p62 protein. Therefore, p62 is commonly considered a late marker of autophagy [48].

**Figure 5:**
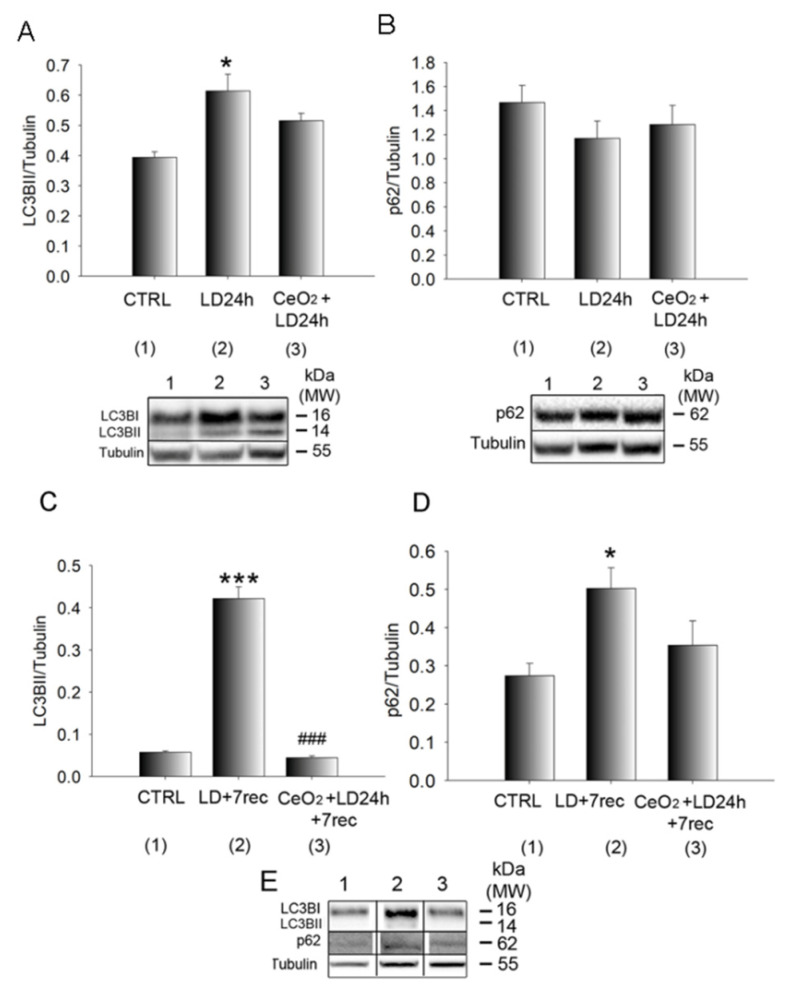
Quantification of the autophagy markers. (**A**,**B**) Western Blot analysis of LC3B-II and p62, respectively, on eye cup samples after 24 h of light exposure. (**C**,**D**) Western Blot analysis of LC3B-II and p62, respectively, on eye cup samples 7 days after light exposure. Statistical analysis was performed by a one-way ANOVA test followed by the Tukey test. Data are shown as mean ± SE (*n* = 4). * *p* < 0.05, *** *p* < 0.001 versus control. ### *p* < 0.001 versus LD. (**E**) Representative Western blot bands of (**C**) and (**D**) panels (1: CTRL, 2: LD + 7 rec, 3: CeO_2_ + LD + 7 rec). The original Western blots are presented in Figure S4. CTRL: Control. LD24h: animals exposed to light damage (1000 lux) for 24 h. LD24h + 7 rec: animals exposed to LD24h followed by 7 days of recovery. CeO_2_ + LD24h: animals treated with cerium oxide nanoparticles and then exposed to LD24h. CeO_2_ + LD24h + 7 rec: animals treated with cerium oxide nanoparticles, then exposed to LD24h and let recover for seven days.

Through Western blot analysis of eye cup samples, we found that LC3B-II was increased as a function of time by LD. It was significantly up-regulated after 24 h of LD compared to the control and its expression decreased after seven days of recovery when compared to the LD for 24 h, but was still higher when compared to the control (Figure S3A). Immediately after light damage, nanoceria did not affect the expression of LC3B-II (Figure 5A) compared to the untreated animals. Moreover, p62 was not altered soon after light damage when compared to the control, and, in the nanoceria-treated group, the levels of p62 were similar to the control (Figure 5B). A more unique result was observed seven days after LD, when the RPE was degenerated as a consequence of light damage. Furthermore, LC3B-II was significantly reduced in the nanoceria-treated animals compared to the untreated ones and its levels were similar to the control group (Figure 5C). p62 levels were significantly increased 7 days after light damage compared to the control and were reduced by nanoceria to levels similar to the control (Figure 5D). Representative Western blot bands of LC3B-II and p62 after 7 days from light damage are shown in Figure 5E.

Moreover, cerium oxide nanoparticles injected in healthy rats did not alter the expression of LC3B-II (Figure S3B).

### 3.6. Nuclear and Cytoplasmic LC3B Localization

**Figure 6:**
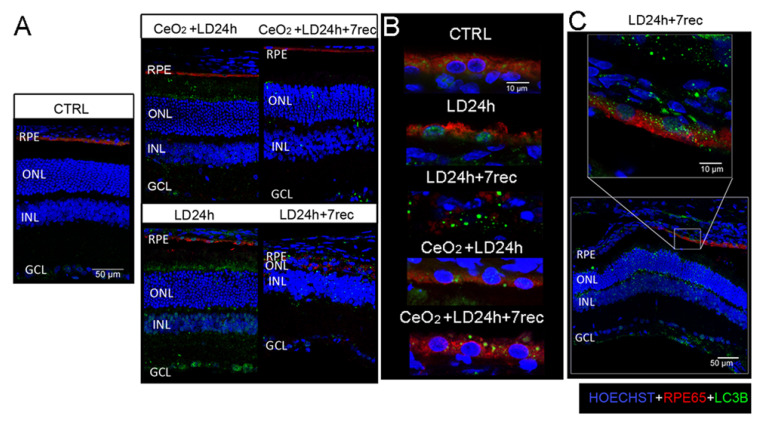
Localization of LC3B on retinal cryosections. (**A**) Representative confocal images of anti-RPE65 (red) and anti-LC3B (green) immunostaining on retinal cryosections of all the experimental groups counterstained with Hoechst (blue), showing the central dorsal area (hot spot) of retinal cryosections crossing the optic nerve. Scale bar: 50 µm. (**B**) Magnification on the RPE layer at the dorsal area of retinal cryosections immunolabeled with anti-RPE65 (red) and anti-LC3B (green) and counterstained with Hoechst (blue). Scale bar: 10 µm. (**C**) Representative confocal image of anti-RPE65 (red) and anti-LC3B (green) immunostaining of the LD24h + 7 rec group showing the region at the edge of the degenerated area (near hot spot) and a magnification on the RPE layer. Scale bars: 50 µm, 10 µm. RPE: retinal pigment epithelium. ONL: outer nuclear layer. INL: inner nuclear layer. GCL: ganglion cell layer. RPE: retinal pigment epithelium. CTRL: control. LD24h: animals exposed to 24 h of light damage. LD24h + 7 rec: animals exposed to 24 h of light damage followed by seven days of recovery. CeO_2_ + LD24h: animals treated with cerium oxide nanoparticles and exposed to 24h of light damage. CeO_2_ + LD24h + 7 rec: animals treated with cerium oxide nanoparticles and exposed to 24 h of light damage, which was followed by seven days of recovery.

We also performed LC3B/RPE65 immunostaining on retinal cryosections (Figure 6). According to the Western blot results, the LC3B signal was progressively higher in all the retinal layers of the LD groups (Figure S5A). The nanoceria-treated animals showed a reduction of the LC3B signal along the entire retina after seven days from LD, while it was abundant in the nanoceria-treated animals immediately after LD (Figure 6A). LC3B signal was easily detectable in RPE65 positive cells at the dorsal region in LD animals (Figure 6B), except for the group LD24h + 7 rec in which the RPE was overly disorganized.

Furthermore, the LC3B signal was localized in the nuclei of the RPE cells in the LD12h (Figure S5B) and LD24h groups, suggesting alterations in the autophagic flux as a consequence of light exposure. LC3B was localized in the nuclei of RPE cells in the nanoceria-treated animals immediately after LD. On the contrary, LC3B was present only in the cytoplasm of the RPE cells of the nanoceria-treated animals seven days after LD as well as in the cytoplasm of the RPE cells of the control group. Since the RPE was not detectable at the centre of the dorsal retina, we looked at the RPE cells limiting the degenerated area in the LD24h + 7 rec group. The LC3B signal was also present in the nuclei of the RPE cells in this region (Figure 6C). Based on these results, indicating that light damage induces both cell death and autophagy alterations in the RPE, we wondered whether an association of these two processes coexisted in this light-damaged model. To answer this question, we performed the TUNEL assay together with the anti-LC3B immunostaining on retinal cryosections of LD animals, where the displacement of LC3B in the nuclei of the RPE cells was highlighted. We found a co-localization of LC3B in TUNEL positive nuclei of the RPE layer (Figure S6). This result suggests that altered autophagy and cell death occurred concomitantly in the RPE of light damaged animals and supports the hypothesis that a crosstalk between apoptosis and autophagy [27] could be involved in the degeneration of RPE cells in our experimental model.

## 4. Discussion

Cerium oxide nanoparticles are synthetic anti-oxidant agents designed to be ROS scavengers with self-renewal auto-regenerative features [35]. Cerium oxide nanoparticles localize in the retina for up to one year [49] and maintain their anti-oxidant properties without exhaustion [34,50]. Moreover, there are controversial data about the toxicity of the nanoceria in the different tissues [51]. However, it was previously demonstrated that nanoceria are safe and biocompatible for the retina [49,52]. On this basis, cerium oxide nanoparticles represent an eligible therapeutic strategy for treating retinal pathologies. They have been extensively studied in a variety of models of retinal degeneration and their neuroprotective effects on photoreceptors are widely known [31]. However, until now, the effects of cerium oxide nanoparticles on retinal pigment epithelium were never investigated and this paper represents an innovative discovery in this field. The RPE plays a pivotal role in the maintenance of a correct retinal homeostasis and any alterations in its structure and function inevitably affect visual function [4]. This role is particularly marked in age-related macular degeneration pathogenesis in which the slow apoptosis of the RPE, due to the concomitance of multiple risk factors such as age, oxidative stress, and lipofuscin/drusen accumulation, culminates in extensive degeneration and retinal atrophy [9].

Based on the pivotal role that an altered RPE plays in the development of AMD, for the first time in this study, cerium oxide nanoparticles were tested in order to understand whether they would be able to protect the RPE in an experimental model of AMD. We demonstrated for the first time that intravitreally injected cerium oxide nanoparticles target the RPE and trigger its protection from light-induced degeneration. Cerium oxide nanoparticles labelled with FITC were found localized in the RPE layer and specifically in the cytoplasm of RPE cells. From this important finding, we can assume that photoreceptor neuroprotection by cerium oxide nanoparticles, highlighted in previous studies in this experimental model [32], is mediated by the structural preservation of the RPE and, thus, it could be the consequence of its protection. In particular, cerium oxide nanoparticles not only inhibited RPE cell death in vitro and in vivo but also prevented the occurrence of cellular stress signs, which is characteristic of the epithelial-mesenchymal transition induced by light damage in the untreated animals. This leads to the maintenance of a correct RPE layer structure. Light damage induced the reduction of the RPE65 protein in the RPE cells at the central hotspot immediately after the exposure, while this reduction was not observed in the RPE of the nanoceria-treated animals. Previous studies showed that RPE65^−/−^ null mice, which lack bleachable visual pigments, are resistant to light damage, which indicates that rhodopsin is a key factor involved in light-induced degeneration [53]. On this basis, it is possible that the reduction of RPE65 expression due to light exposure is a self-protective mechanism put in place by the RPE in order to counteract photo-oxidative damage. On the other hand, RPE65 down-regulation is also a common sign of de-differentiation in the RPE cells [13,45]. Treatment with cerium oxide nanoparticles preserved the expression of RPE65, which indicates the inhibition of the de-differentiation processes. Moreover the protective mechanisms carried out by the treatment couldinterfere with the rhodopsin-mediated damage in the LD model. Even more remarkable, it is the ability of cerium oxide nanoparticles to prevent the breakdown of the RPE layer, which is induced seven days after LD. Moreover, it was previously demonstrated that, seven days after LD, the photoreceptor layer is invaded by blood vessels [38]. Although the origin of those vessels has not yet been clarified, it is possible that they originate from the choroid due to the RPE layer deterioration shown in this study. On this basis, RPE protection by cerium oxide nanoparticles could also allow the prevention of neovascularization in the photoreceptor layer by avoiding the progression toward wet AMD. We also observed that altered RPE cells were still present at the edge of the degenerated area after seven days from LD, and that cerium oxide nanoparticles also allowed the protection of those cells. This observation indicates that degeneration processes were still ongoing in the RPE layer and that RPE cells could continue to die after a longer time from LD. In this context, it will be useful to investigate the ability of cerium oxide nanoparticles to protect the RPE over time.

Another important finding of this study was that nanoceria interfered with the autophagy alterations, which occur in the damaged RPE. Interestingly, autophagy activation, as shown by the up-regulation of the LC3B-II marker [48], was not modulated by the nanoceria treatment immediately after LD. Conversely, it was significantly down-regulated by the treatment seven days after LD. P62, whose accumulation is a hallmark of defective autophagy [48], was not modulated immediately after light damage, but it was increased after seven days and it was reduced by the nanoceria treatment. Furthermore, for the first time, it was demonstrated that LC3B was present in the nuclei of RPE cells after light damage. Nuclear LC3B has been associated with several processes. Some data suggest that it is implicated in the degradation of the nuclear lamina and chromatin, which mediates a nuclear-cytoplasm transport, and this mechanism is associated with cellular senescence [54]. Another study indicated that intra-nuclear autophagy-associated proteins correlate with prolonged survival in cancer cells [55]. Furthermore, this study highlighted that nuclear LC3B co-localized with fragmented DNA in the RPE cells, which suggests that a crosstalk between altered autophagic flux and apoptosis could be implicated, as indicated in other studies [27,56]. Moreover, based on the unchanged LC3B-II levels in treated animals compared to the untreated ones, autophagy activation is likely to be a self-protective mechanism immediately after LD, mainly due to light exposure, as supported by the unaltered p62 amount after LD. In fact, light exposure induces sustained activation of the visual cycle and accelerated metabolism between RPE and photoreceptors, which leads to increased demand of photoreceptor disks renewal. This occurs in RPE cells through a non-canonical autophagy mechanism called LAP (LC3-associated phagocytosis) [23]. Conversely, seven days after LD, LC3B-II and p62 were increased in the untreated animals and a significant RPE and photoreceptors death was observed while the autophagy markers were down-regulated by the nanoceria treatment and the retina was protected. On this basis, it is possible that sustained autophagy after light damage culminates in a defective signal that is involved in retinal degeneration [56]. At the more advanced stage of the damage, autophagy activation was likely not induced by light exposure but by the oxidative stress due to the accumulation of toxic metabolites during light exposure. Oxidative stress is widely considered a major inductor of autophagy activation [30] and, accordingly, ROS accumulation is a peculiar feature of the LD model [57,58]. Cerium oxide nanoparticles reduce oxidative stress as the main mechanism of action in the LD model, as extensively demonstrated [34,59]. Accordingly, in this study, we showed that nanoceria also prevent excessive and sustained autophagy activation, which leads to RPE protection. Based on recent data that indicate an interplay between altered autophagy and EMT [28], we can assume that the attenuation of EMT by cerium oxide nanoparticles highlighted in this study in RPE cells may be exerted by regulating autophagy.

Taken together, the data of this study delineate a therapeutic profile of cerium oxide nanoparticles that, in view of their specific RPE localization and protection, makes them a powerful protective factor for the RPE and treatment for AMD patients. Further studies will be useful to deepen the sub-cellular localization of the nanoparticles, their efficacy on the RPE when administered in ongoing retinal degeneration processes, and in other animal models of AMD. This will allow us to better understand all the mechanisms of action by nanoceria against AMD pathogenesis in order to shorten the gap between bench and bedside and translating this treatment to clinical practice.

## Supplementary Materials

The following are available online at https://www.mdpi.com/2073-4409/9/7/1617/s1. Figure S1: Cell viability assay obtained by crystal violet staining on ARPE-19 cells stressed with H_2_O_2_ at increasing concentrations (200 µM: 400 µM, 600 µM, 800 µM, 1000 µM), Figure S2A: Representative fluorescence images of retinal cryosections, Figure S2B: Number of TUNEL positive cells in the retinal pigment epithelium, Figure S3A: Western Blot analysis of LC3B-II on eye cups from rats exposed to light damage (LD) for different times, Figure S3B: Western Blot analysis of LC3B-II on eye cups from healthy rats intravitreally injected with cerium oxide nanoparticles and untreated control rats, Figure S4: Original whole western blot, Figure S5A: Representative confocal images, Figure S5B: Magnification on the RPE layer, Figure S6: Representative confocal images of anti-LC3B immunostaining and TUNEL assay.
