# Supplementary material for: Nanoceria Particles Are an Eligible Candidate to Prevent Age-Related Macular Degeneration by Inhibiting Retinal Pigment Epithelium Cell Death and Autophagy Alterations
Source: Cells. 2020 Jul 4;9(7):1617. doi: 10.3390/cells9071617 (PMC7408137; doi:10.3390/cells9071617)
Supplement: Supplementary file 1 [file cells-09-01617-s001.pdf]

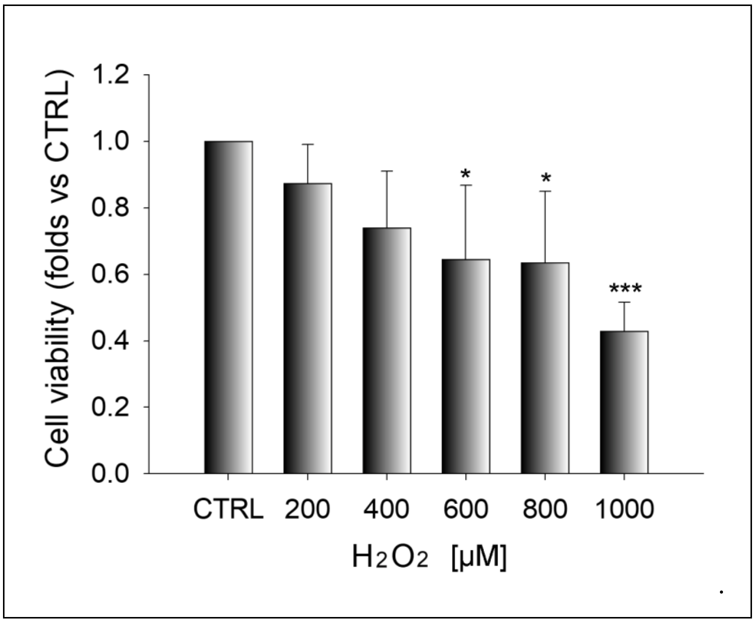

Figure S1: Cell viability assay obtained by crystal violet staining on ARPE-19 cells stressed with H_2O_2 at increasing concentrations (200 μM: 400 μM, 600 μM, 800 μM, 1000 μM). Data are shown as mean ± SE. Statistical analysis was performed by one-way ANOVA followed by Tukey test (***n*** = 4). *$p < 0.05$, **$p < 0.005$, ***$p < 0.0001$ versus CTRL.

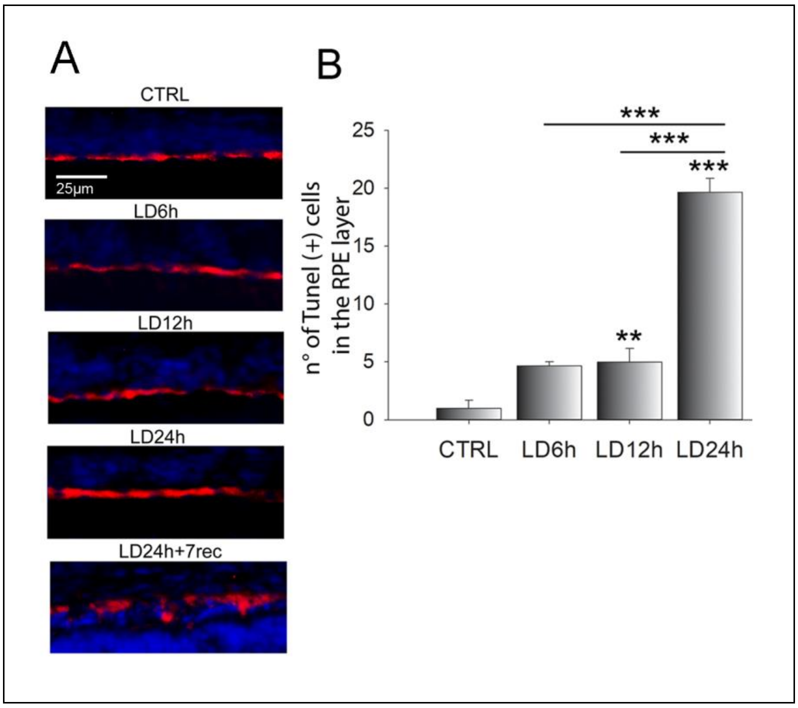

Figure S2 (**A**) Representative fluorescence images of retinal cryosections immunolabelled with anti-RPE65 (red) and counterstained with Hoechst (blue). The images show the central dorsal retina of rats exposed to light damage (LD) for different times. (**B**) Number of TUNEL positive cells in the retinal pigment epithelium counted along the dorsal retina of rats exposed to light damage (LD) for different times. Data are shown as mean ± SE ($n = 3$). * $p < 0.05$; ** $p < 0.01$; *** $p < 0.001$ versus Control. CTRL: Control; LD: light damage; LD24h: animals exposed to light damage (1000 lux) for 24h; LD24h + 7rec: animals exposed to LD24h followed by 7 days of recovery; CeO_2 + LD24h: animals treated with cerium oxide nanoparticles and then exposed to LD24h; CeO_2+LD24h+7rec: animals treated with cerium oxide nanoparticles and then exposed to LD24h and let recover for 7 days.

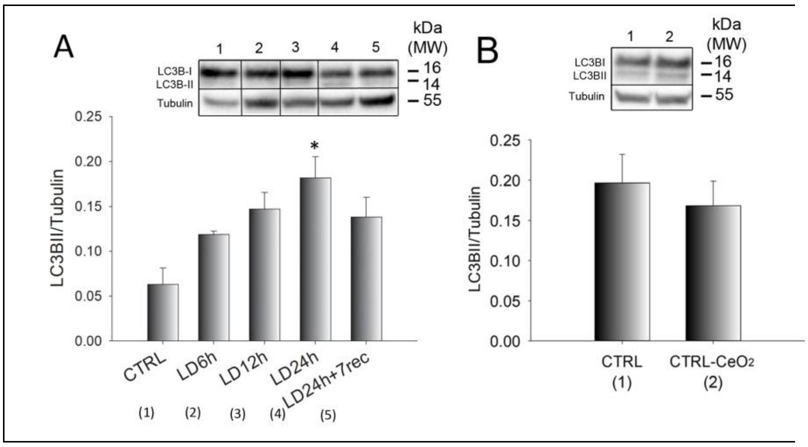

Supplementary Figure S3 (**A**) Western Blot analysis of LC3B-II on eye cups from rats exposed to light damage (LD) for different times. Data are shown as mean ± SE (*n* = 3). * $p < 0.05$; ** $p < 0.01$; *** $p < 0.001$ versus Control (CTRL). (**B**) Western Blot analysis of LC3B-II on eye cups from healthy rats intravitreally injected with cerium oxide nanoparticles and untreated control rats. Data are shown as mean ± SE (*n* = 4). CTRL: Control; LD: light damage; LD24h: animals exposed to light damage (1000 lux) for 24h; LD24h+7rec: animals exposed to LD24h followed by 7 days of recovery; CeO2+LD24h: animals treated with cerium oxide nanoparticles and then exposed to LD24h; CeO_2+LD24h+7rec: animals treated with cerium oxide nanoparticles and then exposed to LD24h and let recover for 7 days.

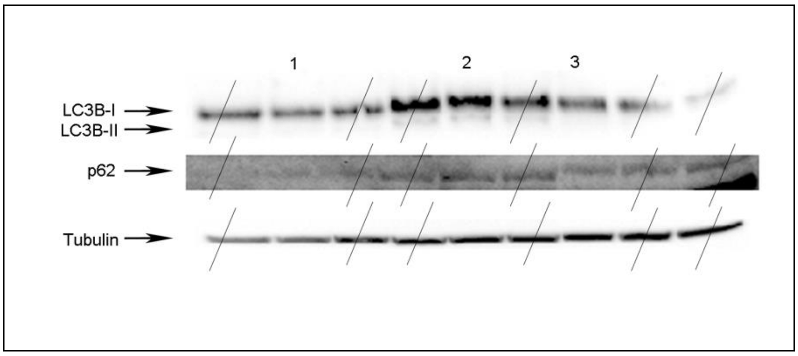

Supplementary Figure S4: Original whole western blot. For the final figure, the Adobe Photoshop software was used to flip the western blot horizontally and crop the horizontal line of LC3BI/LC3BII, p62 and tubulin bands indicated by the arrows. They were then assembled in Figure 5E.

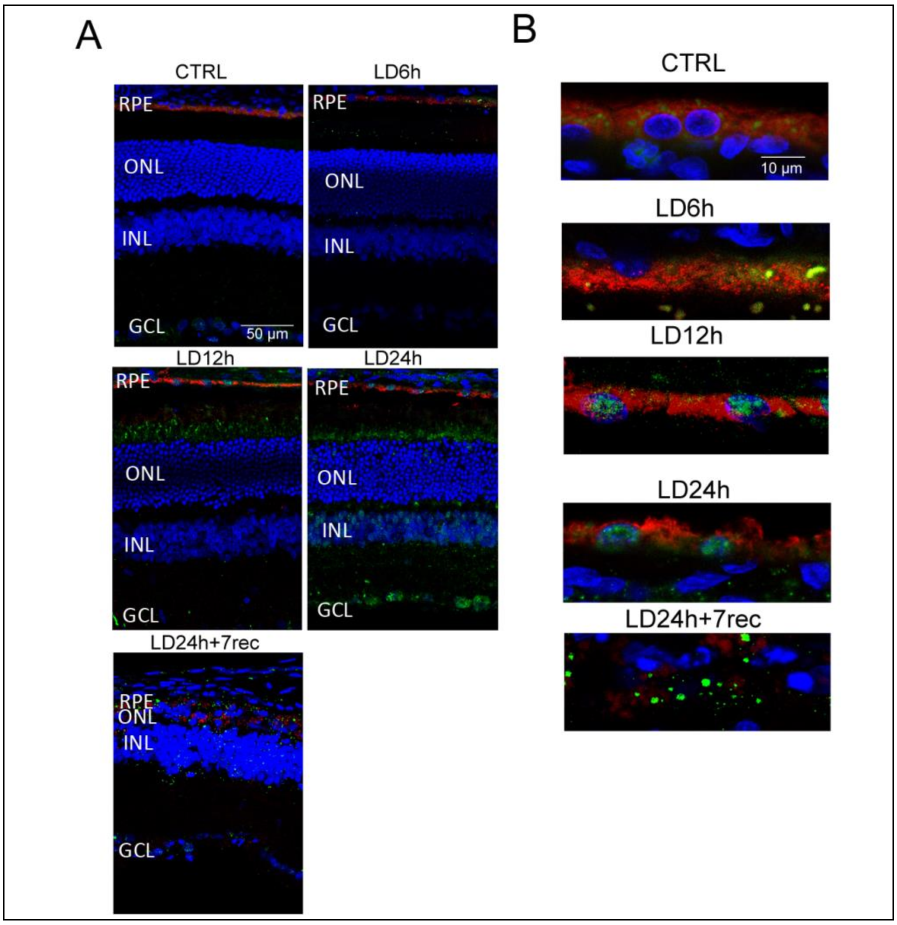

Figure S5 (**A**) Representative confocal images of anti-RPE65 (red) and anti-LC3B (green) immunostaining on retinal cryosections of the LD time course counterstained with Hoechst (blue), showing the central dorsal area (hot spot) of retinal cryosections crossing the optic nerve. Scale bar:50µm. (**B**) Magnification on the RPE layer at the dorsal area of retinal cryosections immunolabeled with anti-RPE65 (red) and anti-LC3B (green) and counterstained with Hoechst (blue). The images are representative of the LD time course. Scale bar:10µm. RPE:retinal pigment epithelium; ONL: outer nuclear layer; INL: inner nuclear layer; GCL: ganglion cell layer.RPE:retinal pigment epithelium; CTRL:control; LD24h: animals exposed to 24h of light damage; LD24h+7rec: animals exposed to 24h of light damage followed by 7 days of recovery..

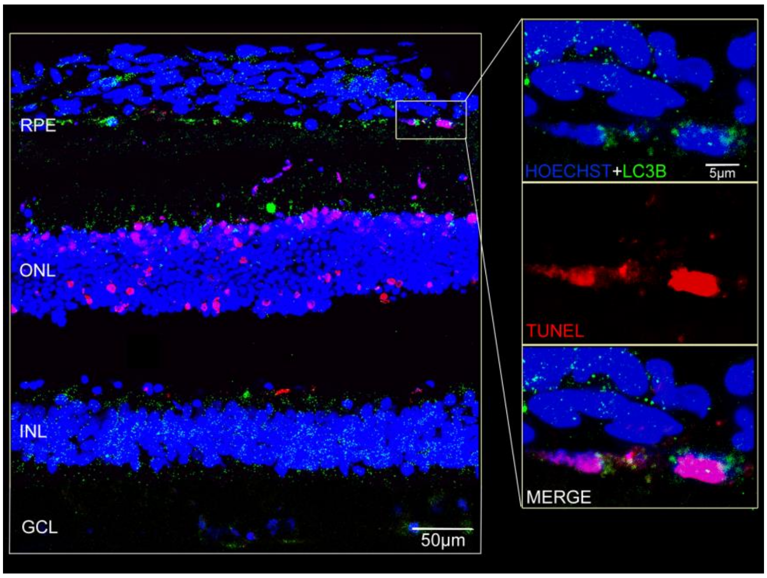

Figure S6: Left: Representative confocal images of anti-LC3B (green) immunostaining and TUNEL assay (red) on retinal cryosections after 24h of light damage time course counterstained with Hoechst (blue). Scale bar: 50µm.Right: High magnifications of the image in the white frame showing the co-localization of nuclear LC3B with TUNEL positive nuclei. Scale bar:5 µm.
